# The Impact of mHealth Interventions on Breast Cancer Awareness and Screening: Systematic Review Protocol

**DOI:** 10.2196/resprot.8043

**Published:** 2017-12-21

**Authors:** Temitope O Tokosi, Jill Fortuin, Tania S Douglas

**Affiliations:** ^1^ Division of Biomedical Engineering Department of Human Biology University of Cape Town Cape Town South Africa

**Keywords:** mHealth, breast cancer, women, awareness, screening

## Abstract

**Background:**

Mobile health (mHealth) is the use of mobile communication technologies to promote health by supporting health care practices (eg, health data collection, delivery of health care information). mHealth technologies (such as mobile phones) can be used effectively by health care practitioners in the distribution of health information and have the potential to improve access to and quality of health care, as well as reduce the cost of health services. Current literature shows limited scientific evidence related to the benefits of mHealth interventions for breast cancer, which is the leading cause of cancer deaths in women worldwide and contributes a large proportion of all cancer deaths, especially in developing countries. Women, especially in low- and middle-income countries (LMICs), are faced with low odds of surviving breast cancer. This finding is likely due to multiple factors related to health systems: low priority of women’s health and cancer on national health agendas; lack of awareness that breast cancer can be effectively treated if detected early; and societal, cultural, and religious factors that are prevalent in LMICs. The proposed systematic review will examine the impact of mHealth interventions on breast cancer awareness and screening among women aged 18 years and older.

**Objective:**

The objectives of this study are to identify and describe the various mHealth intervention strategies that are used for breast cancer, and assess the impact of mHealth strategies on breast cancer awareness and screening.

**Methods:**

Literature from various databases such as MEDLINE, EMBASE, PsycINFO, CINAHL, and Cochrane Central Register of Controlled Trials will be examined. Trial registers, reports, and unpublished theses will also be included. All mobile technologies such as cell phones, personal digital assistants, and tablets that have short message service, multimedia message service, video, and audio capabilities will be included. mHealth is the primary intervention. The search strategy will include keywords such as “mHealth,” “breast cancer,” “awareness,” and “screening,” among other medical subject heading terms. Articles published from January 1, 1964 to December 31, 2016 will be eligible for inclusion. Two authors will independently screen and select studies, extract data, and assess the risk of bias, with discrepancies resolved by dialogue involving a third author. We will assess statistical heterogeneity by examining the types of participants, interventions, study designs, and outcomes in each study, and pool studies judged to be statistically homogeneous. In the assessment of heterogeneity, a sensitivity analysis will be considered to explore statistical heterogeneity. Statistical heterogeneity will be investigated using the Chi-square test of homogeneity on Cochrane's Q statistic and quantified using the I-squared statistic.

**Results:**

The search strategy will be refined with the assistance of an information specialist from November 1, 2017 to January 31, 2018. Literature searches will take place from February 2018 to April 2018. Data extraction and capturing in Review Manager (RevMan, Version 5.3) will take place from May 1, 2018 to July 31, 2018. The final stages will include analyses and writing, which is anticipated occur between August 2018 and October 2018.

**Conclusions:**

The knowledge derived from this study will inform health care stakeholders, including researchers, policy makers, investors, health professionals, technologists, and engineers, on the impact of mHealth interventions on breast cancer screening and awareness.

**Trial Registration:**

Prospero registration number CRD42016050202

## Introduction

Mobile health (mHealth) is a component of electronic health (eHealth) and involves the use of mobile communication technologies to promote health by supporting health care practices (eg, health data collection, delivery of health care information, or patient observation and provision of care) [1]. mHealth is considered to have a positive impact on health systems through improved access to and quality of health care, as well as reduction in the cost of health services [2]. In addition, mHealth can facilitate continuous health monitoring individually and at the population level. mHealth has the potential to support chronic disease self-management, reduce the number of patient visits to health care facilities, and provide personalized, localized, and on-demand interventions [3,4].

The mobile devices or technologies applied to health include mobile phones as well as smartphones, tablets, portable media players, and their mHealth apps [5]. The growing enthusiasm for mHealth is driven not only by its demonstrated benefits, but also by the widespread availability of mobile phones, and the relatively low levels of literacy required to use them [1]. mHealth technologies enable timely collection, transmission, storage, and transformation of data, as well as data analyses and automated reporting [1]. The modes of communication of these technologies take the form of short message service (SMS), instant messaging, voice calls, social media, the Internet, and email [1,6].

mHealth applications also contribute to improvements in health care through diagnoses, monitoring, and/or treatment. One application developed for use in treatment is *WE-CARE*, which is an intelligent telecardiology system using mobile 7-lead electrocardiogram devices [7,8]. Another mHealth application example known as *PIERS on the Move* is a low-cost, easy-to-use application for accurately predicting the risk of adverse outcomes associated with preeclampsia in pregnant women [9]. *MEDITECH* allows access to clinical data including lab results, vital signs, intake and output, allergies, active medications, and documents (reports and notes) on a smartphone [9]. *Wireless System for Emergency Responders* is an application for emergency medical service specialists that identifies chemical and biological hazards on the basis of symptoms and signs by providing access to the National Library of Medicine (NLM) Hazardous Substances Data Bank radiological and biological substance report [10].

Breast cancer is the most common cancer in women worldwide, with an estimated 1.67 million new cases diagnosed in 2012 [11]. Most women diagnosed in high-income counties are likely to survive this disease, whereas those in low- and middle-income countries (LMICs) are faced with odds of survival as low as 10-25% [11]. This result is likely due to multiple factors related to health systems: low priority of women’s health and cancer on national health agendas; lack of awareness that breast cancer can be effectively treated if detected early; and societal, cultural, and religious factors that are prevalent in LMICs [11]. Breast cancer mortality can be dramatically reduced via screening and early detection, especially in LMICs that have high mortality rates [11]. Male breast cancer is rare but exists, with approximately 2000 men diagnosed with breast cancer per year in the United States [12]. Male patients are slightly older at presentation but their cancers show similarities to female breast cancer [12].

Evidence has shown that intervention by health care practitioners can be effective in improving awareness, knowledge, attitudes, and screening practices for early detection of breast cancer among women [13]. Current literature shows insufficient scientific evidence related to the benefits of mHealth interventions for breast cancer [5,14-16]. Available studies involving mHealth strategies for breast cancer-related interventions have addressed breast health promotion, clinical breast examination, patient navigation, and breast cancer education [11,14]. A systematic analysis of breast cancer-related apps has found that their most common purposes are education, behavior change, fundraising, and advocacy [17].

Several mHealth intervention strategies have been implemented to address breast cancer. One example is an investigation of the effect of mHealth with pedometer use on function and quality of life (QOL) among breast cancer patients [18]. The results reported in this study showed improvements in these parameters [18]. QOL in breast cancer survivors was also shown to be improved by an imagery-based group behavioral intervention for breast cancer survivors using telemedicine [19]. Quintiliani et al demonstrated the feasibility of an mHealth-supported behavioral counselling intervention among breast cancer survivors and found some positive physiological and behavioral changes [20]. A patient-centered, Web- and mobile-based educational and behavioral mHealth intervention strategy has been shown to assess patients’ lymphedema symptoms with high reliability and validity, and was able to enhance self-care strategies for lymphedema symptom management [21]. mHealth has also been used for general cancer applications. An example is the study by Banas et al who developed a peer-to-peer mHealth tool, which is culturally tailored to Spanish speakers (available in Spanish), and connects cancer patients with survivors [22]. In Nigeria, mobile phones were used as tools for improving cancer care by developing helplines for cancer information related to prevention, treatment, and palliative care [23].

Current evidence supports the use of mHealth for addressing cancer broadly. Our intention is to systematically review the literature to aid us in assessing the impact of mHealth on breast cancer screening and awareness. The study examines the impact of mHealth technology as an intervention on breast cancer awareness and screening attendance of women, compared to conventional interventions that promote awareness and screening.

## Methods

This manuscript adheres to the Preferred Reporting Items for Systematic Reviews and Meta-Analyses-Protocols (PRISMA-P) 2015 checklist as a condition of submission for systematic review protocols [24]. This protocol has been registered with PROSPERO, the International Database of Prospective Register of Systematic Reviews, with registration number CRD42016050202 [25].

### Study Design

The study design for this review will include randomized and nonrandomized studies. Nonrandomized studies will include case-control, cohort, and cross-sectional studies in which mHealth was the primary intervention used for breast cancer awareness and screening.

### Study Participants

Study participants will be women aged 18 years and older. All races, ethnicities, employment statuses, occupations, and roles of women in the reviewed studies will be eligible for inclusion.

### Types of Interventions

The relevant mHealth interventions to be included in the study should focus primarily on positively impacting breast cancer awareness and screening. mHealth interventions for health care consumers (women >18 years of age) have been designed to increase healthy behaviors (eg, to increase breast cancer awareness) or increase hospital attendance (eg, increasing the participation of hospital workshops by women to improve their understanding of early breast cancer detection, management, and treatment thereof) [3]. The interventions will be described using the Free et al strategy that categorizes by device (mobile phone, personal digital assistant [PDA]) and modality (eg, SMS, text messaging, multimedia message service [MMS], video) [26].

### Types of Technology

The technology used for mHealth includes mobile devices that have cellular communication capabilities that allow for wireless and/or 3G/4G capabilities. These devices include: mobile phones (including Android, smart, and feature phones), PDAs and PDA phones, tablets, mobile/smart phone apps, ultra-portable computers, and smart books [3,5,26].

The mHealth functions that will be considered comprise voice over Internet protocol (VOIP), SMS, text messaging, MMS, email, social media, and Internet [27]. Some mHealth projects have used single or multiple applications employing one or more mobile phone functions–such as voice, SMS, MMS, and interactive voice response (IVR)–to accomplish mHealth-related tasks [1]. Common applications using various mHealth modalities include: client education and behavior change communication (SMS, MMS, IVR, audio, video, images); sensors and point of care devices (mobile phone camera); registries and vital event tracking (SMS, voice communication); data collection and reporting (voice communication, SMS); electronic health records (mobile Web); electronic decision support (mobile web, IVR); provider communication (SMS, MMS, mobile phone camera); provider work planning and scheduling (SMS, mobile phone calendar); provider training and education (SMS, MMS, IVR, audio, video); human resource management (voice communication, SMS); supply chain management (global positioning system, SMS); and financial transactions and incentives (mobile banking service, airtime transfers) [5,28,29].

### Outcomes

The impact of mHealth interventions on breast cancer awareness and screening will be assessed by reviewing: (1) increased attendance at breast cancer clinics; (2) the stage of breast cancer when diagnosed, as this would assist in determining whether mHealth has promoted early detection and screening; and (3) increased breast cancer enquiries via call centers, online forums, and social media.

User acceptability will not be assessed as an outcome. Breast cancer awareness in this study is described as the ability to be fully informed and knowledgeable of the breast cancer disease [30]. In addition, screening is a system of checking for the presence or absence of a disease (in this study, breast cancer). In situations where actual numbers of women cannot be determined, the baseline for assessment will be determined by the keywords “increase” or “improvement” used in the study. The same criteria will apply to women visiting hospitals for breast cancer screening.

### Study Setting

The setting of the study will not be limited by geographic location. All continents, countries, and health facilities where mHealth research on breast cancer was conducted will be included. This approach allows for all relevant information sources to be captured.

### Exclusion Criteria

The following study types will be excluded from the review: (1) all studies on male breast cancer, due to the rarity of this disease in men; (2) breast cancer detection and treatment studies; (3) studies not performed on human subjects; (4) studies reported before January 1, 1964 and after December 31, 2016; (5) letters, reviews, commentaries, and editorials; (6) studies lacking primary data and/or explicit method description; (7) non-English studies and publications; and (8) duplicate studies that are published in more than one report (the most comprehensive and up-to-date version will be used).

### Search Strategy

While the earliest keyword “mobile health” was identified in the literature in 1991 by Casson and Leder [31], relevant literature will be identified from 1964. This start date corresponds to the earliest identifiable mention of the keyword “telemedicine” in a preliminary search of all the major databases such as Medical Literature Analysis and Retrieval System Online (MEDLINE), Excerpta Medica dataBASE (EMBASE), Psychological Information Database (PsycINFO), Cumulative Index to Nursing and Allied Health Literature (CINAHL), The Cochrane Library (Cochrane Database of Systematic Reviews, Cochrane Central Register of Controlled Trials, Cochrane Methodology Register), National Health Services Health Technology Assessment Database, Web of Science, and Google scholar. The language of publication will be limited to English for reasonable analysis purposes.

Other databases to be considered within the study scope and objectives will include trial registers, SpringerLink, Wiley InterScience, Institute of Electrical and Electronics Engineers, Association for Computing Machinery Digital Library; and CiteSeer [29]. For trial registers, the authors will identify ongoing studies and recently completed trials. The studies to be included will be selected using predefined search terms adapted for the databases to be used. The authors will adapt the experimental findings proposed by Fortuin et al [5] in identifying accurate search terms in the development of an optimum search strategy.

The search strategy will include key terms, as detailed in Table 1 (MEDLINE format). This format was developed with the assistance of a library sciences specialist and will be adapted to all other databases to be searched. A preliminary search was conducted and the number of identified references is reported in Table 1.

Manual searches of reference lists of primary studies included in the review, and the reference lists of relevant and previously published reviews, will be undertaken. Full text articles of the studies extracted from the reference lists will be reviewed. Unpublished studies will be identified from universities and other databases, and the same eligibility criteria will be applied.

### Study Selection

The first author will retrieve all the relevant articles from the various databases, based on the finalized search strategy. All of the literature obtained will be saved using reference management software. Two contributing authors will independently screen the titles and abstracts of retrieved studies for eligibility. These authors will make a final assessment for inclusion using the full text article, and discrepancies and disagreements will be resolved by the third author.

### Data Extraction

A data extraction form will be used for the extraction of key information, including: (1) country of study setting; (2) type of participant/study population/demographic characteristics (eg, women aged 18 years and older); (3) type of mHealth device used (eg, mobile phones, PDAs, smartphones, tablets); (4) method of communication (eg, voice call, SMS, MMS, Unstructured Supplementary Service Data and Web); (5) nature of the mHealth intervention; (6) type of study (ie, study design); (7) type of outcomes measured; and (8) findings/results.

If discrepancies are identified when extracting data, these will be discussed by the first two authors. If no consensus is reached, the third author will mediate. Missing data will be requested from study authors via email [3]. If we receive no response from study authors, an attempt will be made to impute missing standard deviations or standard errors using data from other similar studies in the review, utilizing similar methods and sample sizes, as recommended by Wiebe et al [32].

**Table 1:** A preliminary search query classification.

Number	Query	Items
#1	Search (((((((((((mHealth) OR telemedicine) OR wireless technology) OR mobile phone) OR smartphone) OR cell phone) OR mobile technology) OR mobile device) OR mobile-based phone) OR tablet computer) OR IPAD) OR pda) OR mHealth application Filters: Publication date from 1964/01/01 to 2016/12/31	65,265
#2	Search (((((((((((((((mHealth) OR telemedicine) OR wireless technology) OR mobile phone) OR smartphone) OR cell phone) OR mobile technology) OR mobile device) OR mobile-based phone) OR tablet computer) OR IPAD) OR pda) OR mHealth application)) AND ((voice calling) OR VOIP) OR sms) OR mms) OR texting) OR social media) OR Internet) OR IVR) OR video) OR images)) AND (((voice calling) OR VOIP) OR sms) OR mms) OR texting) OR social media) OR Internet) OR IVR) OR video) OR images Filters: Publication date from 1964/01/01 to 2016/12/31))	211,700
#3	Search (((((((((((((mHealth) OR telemedicine) OR wireless technology) OR mobile phone) OR smartphone) OR cell phone) OR mobile technology) OR mobile device) OR mobile-based phone) OR tablet computer) OR IPAD) OR pda) OR mHealth application)) AND (breast cancer) OR neoplasm Filters: Publication date from 1964/01/01 to 2016/12/31)	421
#4	Search (((((((((((((((mHealth) OR telemedicine) OR wireless technology) OR mobile phone) OR smartphone) OR cell phone) OR mobile technology) OR mobile device) OR mobile-based phone) OR tablet computer) OR IPAD) OR pda) OR mHealth application)) AND (breast cancer) OR neoplasm Filters: Publication date from 1964/01/01 to 2016/12/31))) AND (((((awareness) OR education) OR promotion)) Filters: Publication date from 1964/01/01 to 2016/12/31	88
#5	Search ((((((((((((((((mHealth) OR telemedicine) OR wireless technology) OR mobile phone) OR smartphone) OR cell phone) OR mobile technology) OR mobile device) OR mobile-based phone) OR tablet computer) OR IPAD) OR pda) OR mHealth application)) AND (breast cancer) OR neoplasm Filters: Publication date from 1964/01/01 to 2016/12/31))) AND (((awareness) OR education) OR promotion)) AND ((screening) OR diagnosis) Filters: Publication date from 1964/01/01 to 2016/12/31	52

### Assessing Risk of Bias

Two authors will use the recommendations by the International Cochrane Collaboration [8] to independently assess the risk of bias. These criteria include randomization sequence generation, treatment allocation concealment, blinding of participants, incomplete outcome data, selective outcome reporting, and other sources of bias. All included studies will be scored for bias using these criteria. A descriptive summary for each scoring will be recorded. Discrepancies between the review authors regarding the risk of bias in particular studies will be resolved by dialogue, with involvement of a third author if necessary [3].

### Data Analyses

A descriptive synthesis will be undertaken in accordance with the Centre for Reviews and Dissemination [33]. The characteristics of included studies will be summarized using text and tables, which will include the key data extraction elements (eg, study setting, authors, journal, and study type).

Mean differences and standard deviations will be calculated for continuous outcomes. Ratios and their corresponding 95% confidence intervals will be determined for dichotomous outcomes. Participants, interventions, and outcomes of each study will be examined to ascertain heterogeneity. Data will be pooled; if the collected data is sufficiently similar, a meta-analysis will be conducted. However, if the variability between studies is high the results will not be pooled and a narrative synthesis will take place.

The statistical software Review Manager (RevMan, Version 5.3) will be utilized to capture the data and perform the meta-analysis. The statistical test for heterogeneity includes the I-squared (I^2^) test which quantifies heterogeneity; this test will allow for the quality of the evidence to be validated [8]. When appropriate, a subgroup analysis will be used to determine if varying mHealth applications have an impact on breast cancer awareness and screening among women, and in what context this occurs. Subgroups to be considered for this analysis will include age grouping and geographical region.

Various sensitivity analyses will be undertaken. The first sensitivity analysis will be conducted based on the study quality (risk of bias and level of participant dropout) to investigate possible sources of heterogeneity. The second analysis will determine how excluded studies could have influenced the overall result. The third analysis will be to determine how the result would differ should only high-quality studies be included [8].

## Results

The sourcing of literature in accordance with the inclusion and exclusion criteria of the study is underway. All data extracted are grouped under the various headings as specified in the data extraction form (eg, country of study setting, study population, mHealth device used, communication method, nature of mHealth intervention, type of study, outcomes and findings). It is anticipated that the review will be completed in mid-2018.

## Discussion

This review will identify and describe the impact of mHealth interventions for breast cancer awareness and screening on women of aged 18 years and older. The findings of the systematic review will inform the design of mHealth interventions for breast cancer. Furthermore, the study will highlight which mHealth technology modalities (eg, SMS) are appropriate for the target audience when creating awareness of breast cancer.

### Availability of Data and Material

The datasets used and/or analyzed during the current study will be available from the corresponding author upon reasonable request.

## Abbreviations

eHealth: electronic health
IVR: interactive voice response
LMIC: low- and middle-income country
mHealth: mobile health
MMS: multimedia message service
NRF: National Research Foundation
NLM: National Library of Medicine
PDA: personal digital assistant
QOL: quality of life
SMS: short message service
VOIP: voice over Internet protocol
